# Supplementary figures and images for: Primary complex motor stereotypies are associated with de novo damaging DNA coding mutations that identify KDM5B as a risk gene
Source: PLoS One. 2023 Oct 3;18(10):e0291978. doi: 10.1371/journal.pone.0291978 (PMC10547198; doi:10.1371/journal.pone.0291978)

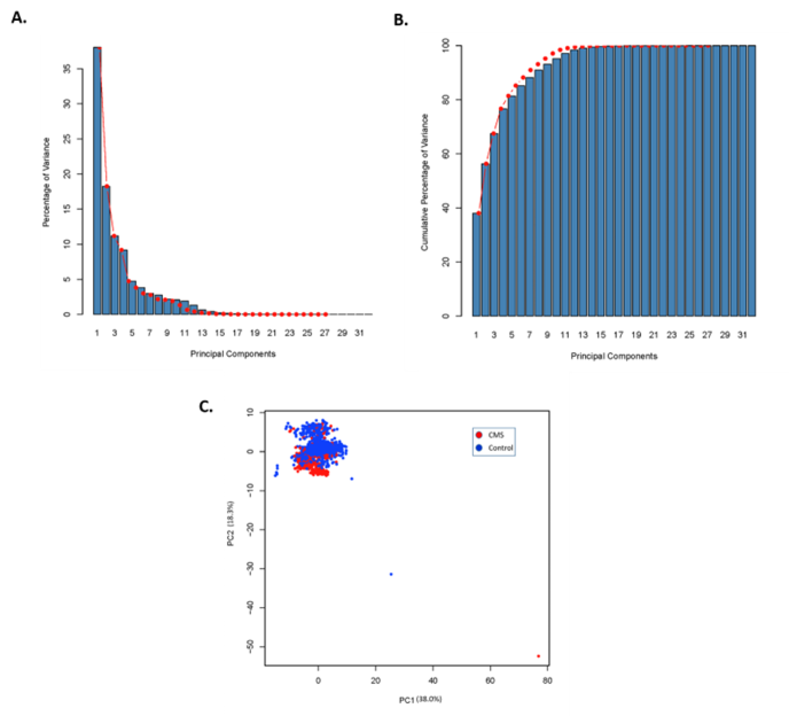

Supplement: S1 Fig — Scree plots following Principal Components Analysis (PCA), showing (A) the percentage of variance captured by each of the first 32 principal components, and (B) the cumulative percentage of variance captured by these same components in the exome metrics data from cases and controls. The “elbow” of the scree plot is visualized to be around the 5th principal component. This was confirmed by the Factominer R code function “estim_ncp()”. The first 5 PCs capture over 80% of the variance, and this number of PCs was used to determine PCA outliers during quality control (see S1 Table and S1 File). (C) Individual plots for the first two principal components, based on PCA of exome sequencing quality metrics. pCMS cases are plotted in red, and controls in blue. The first two PCs together capture 56.3% of the variance. R code to generate this data and figure are in S1 File, and individual PC factor values are in S1 Table. This figure includes PCA outliers (>3 standard deviations from the mean in PCs 1–5), which were removed during quality control, prior to further analysis of case-control data. (TIF) [file pone.0291978.s001.tif]

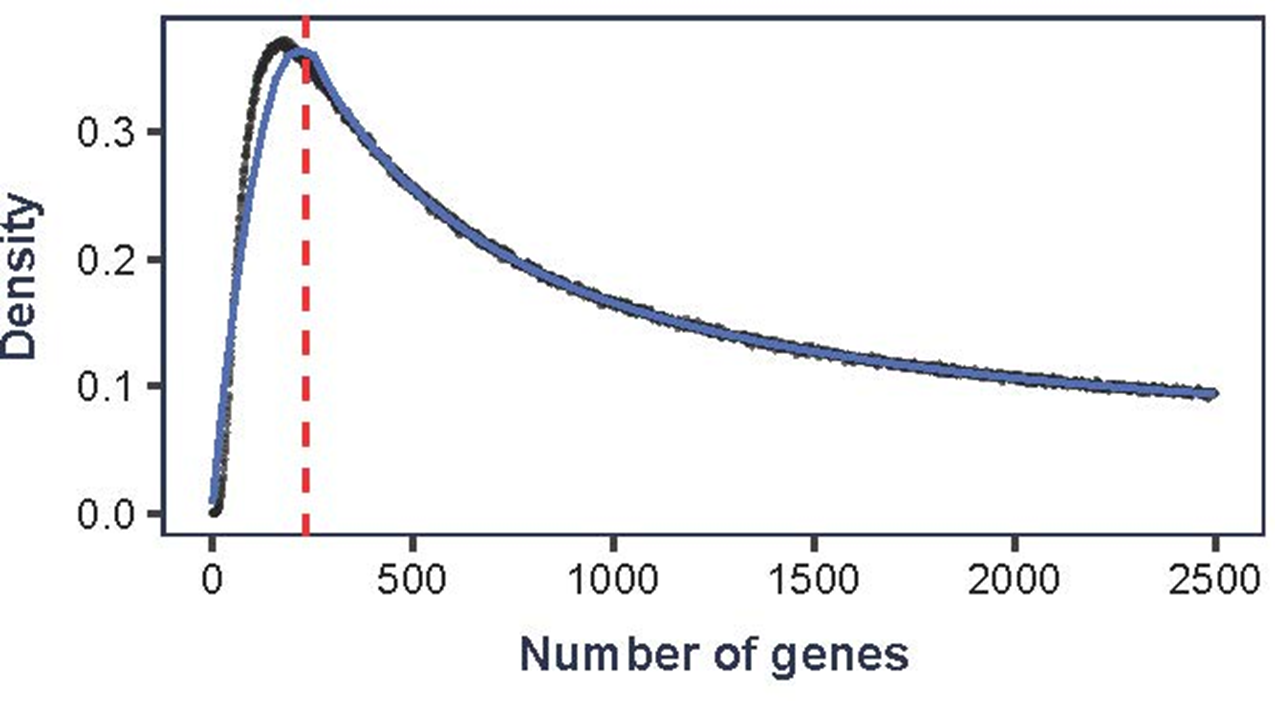

Supplement: S2 Fig — For each number of possible risk genes between 1–2,500, we conducted 50,000 simulations to determine the number of risk genes that yielded the closest agreement between our observed and simulated data. This MLE method yields an estimate of 184 pCMS risk genes (red vertical line). See S1 File. (TIF) [file pone.0291978.s002.tif]

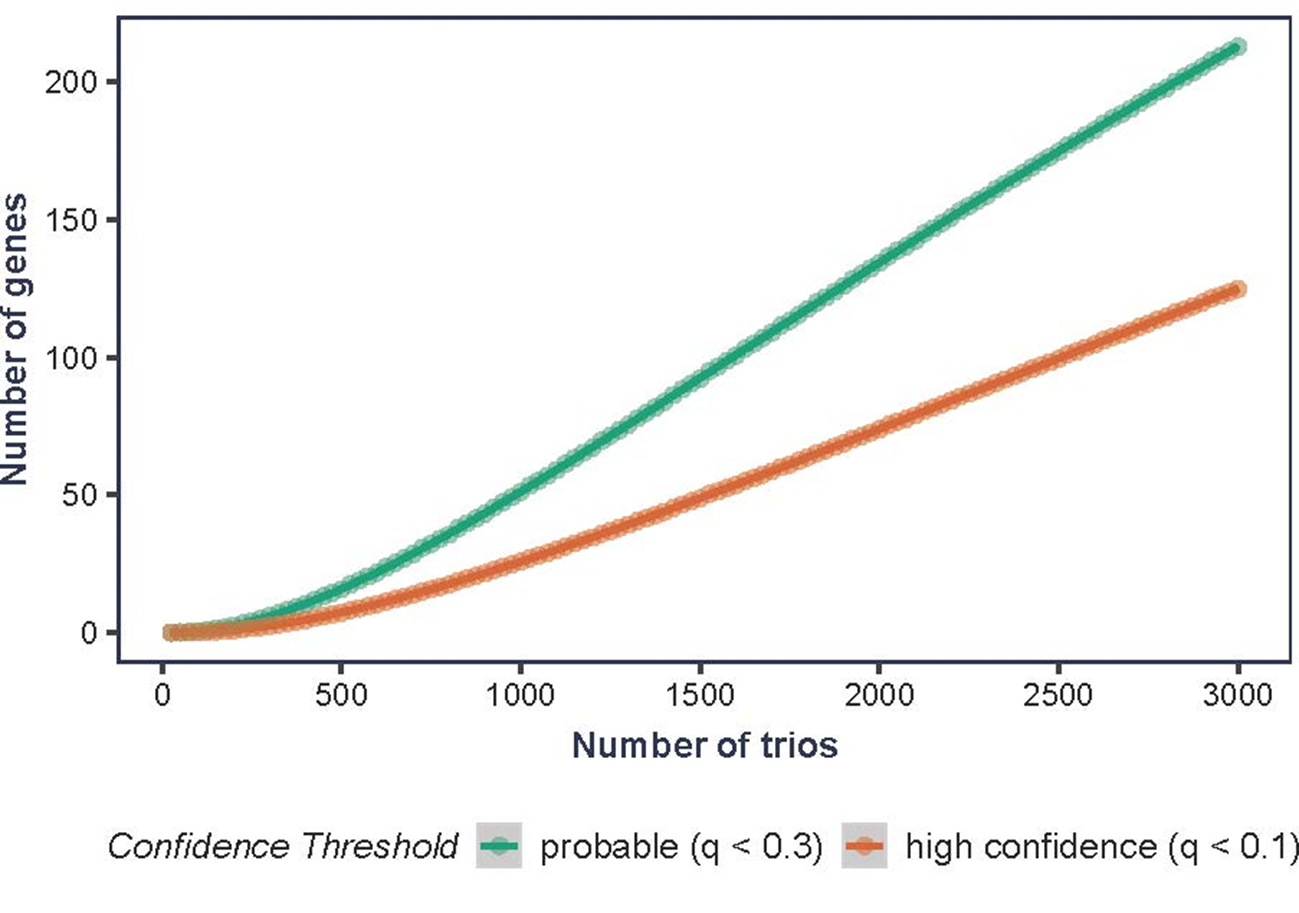

Supplement: S3 Fig — Using our estimate of 184 risk genes (based on the MLE method–see Main Text and S1 File), we estimated the number of probable (FDR q<0.3) and high-confidence (FDR q<0.1) risk genes that will be discovered as more pCMS trios are sequenced. We performed 10,000 simulations at each cohort size from 25–3,000 trios, randomly generating variants and assigning to risk genes in agreement with the proportions seen in our data, then applying the TADA-Denovo algorithm. Based on these simulations, WES of 500 trios should find 16 probable and 7 high-confidence risk genes; 1000 trios should find 51 probable and 26 high-confidence risk genes. (TIF) [file pone.0291978.s003.tif]

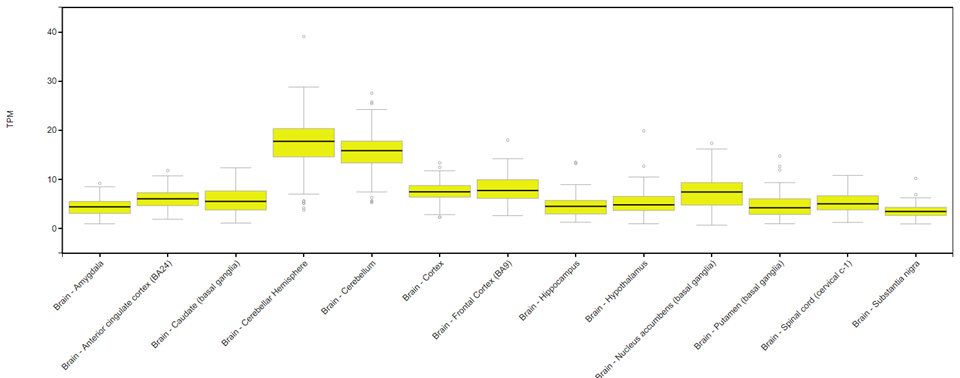

Supplement: S4 Fig — Brain expression by region for KDM5B. Data is from GTEx Analysis Release V7 (dbGaP Accession phs000424.v7.p2) (https://gtexportal.org/home/gene/KDM5B). Expression values are shown in Transcripts Per Million (TPM), calculated from a gene model with isoforms collapsed to a single gene. No other normalization steps have been applied. Box plots are shown as median, 25th, and 75th percentiles. Points are displayed as outliers if they are above or below 1.5 times the interquartile range. Further details about expression quantification and samples can be found at https://gtexportal.org/home/documentationPage#AboutData. (TIF) [file pone.0291978.s004.tif]

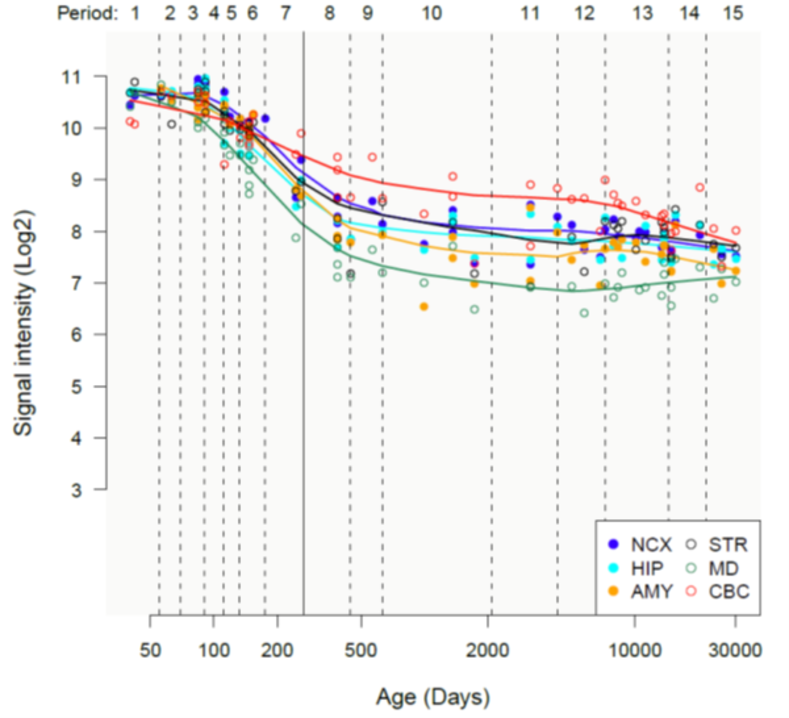

Supplement: S5 Fig — Brain expression trajectories of KDM5B in the developing human brain. Expression data is from the Brainspan Consortium (brainspan.org, hbatlas.org), generated using the Affymetrix GeneChip Human Exon 1.0 ST Array platform. Vertical axis is the log2-transformed array signal intensity, which is proportional to transcript expression. A stringent threshold of ≥6 was required to meet criteria for brain expression. Horizontal axis represents periods of human development and adulthood as previously defined by Kang et al (2011). Birth begins period 8 and adolescence begins period 12. Brain regions are by color: neocortex (NCX), hippocampus (HIP), amygdala (AMY), striatum (STR), mediodorsal nucleus of the thalamus (MD), cerebellar cortex (CBC). (TIF) [file pone.0291978.s005.tif]

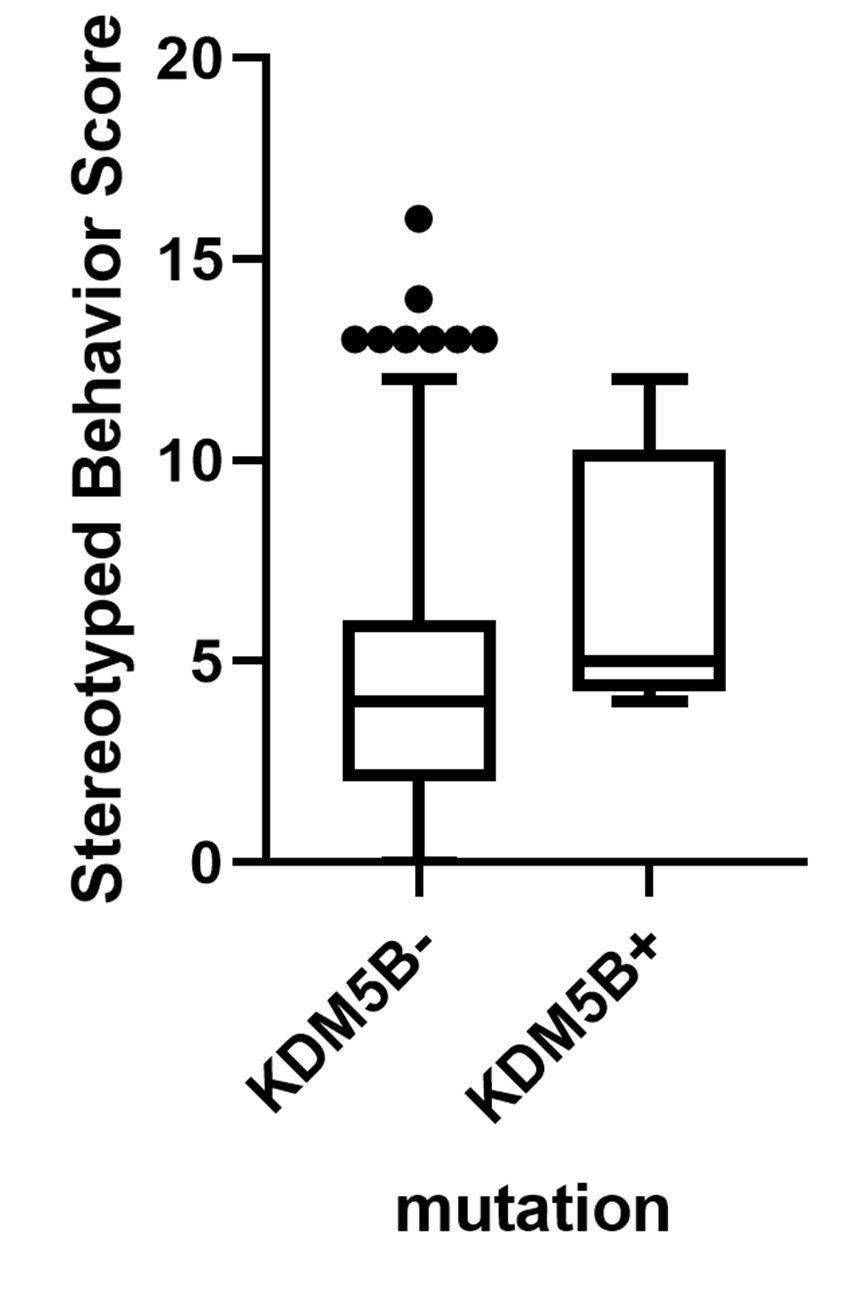

Supplement: S6 Fig — Tukey box and whisker plot of Stereotyped Behavior Score (SBS) from the RBS-R (Repetitive Behavior Scale-Revised) in aged-matched Simons Simplex Collection ASD probands with (+, n = 4) and without (-, n = 364) de novo damaging mutations in KDM5B. Two-tailed Mann-Whitney test of ages (months) between groups: p = 0.86. One-tailed Mann-Whitney test of SBS between groups: p = 0.076. (TIF) [file pone.0291978.s006.tif]
