# Supplementary material for: Primary complex motor stereotypies are associated with de novo damaging DNA coding mutations that identify KDM5B as a risk gene
Source: PLoS One. 2023 Oct 3;18(10):e0291978. doi: 10.1371/journal.pone.0291978 (PMC10547198; doi:10.1371/journal.pone.0291978)
Supplement: S1 File — (DOCX) [file pone.0291978.s012.docx]

# SUPPLEMENTARY METHODS

## Subjects and Assessment Measures

The Stereotypy Severity Scale (SSS) is a 5-item caregiver questionnaire consisting of two components (Motor and Impairment) for the ranking of motor stereotypy severity (1). The SSS Motor score (range: 0-18) quantifies motor severity and rates movements along four discriminate dimensions: number (0-3), frequency (0-5), intensity (0-5), and interference (0-5). The SSS Impairment score (range 0-50) is an independent rating of difficulties in self-esteem, family, school, or social acceptance caused by the movements.

The ASSQ is a 27-item caregiver questionnaire addressing symptoms of ASD (2).

Other parent-completed measures included: the Multidimensional Anxiety Scale for Children (MASC), assessing symptoms of anxiety (3); the ADHD-Rating Scale-IV (4) and Conners Parent Rating Scale (CPRS), assessing symptoms of ADHD; the Child Yale Brown Obsessive-Compulsive Scale (CY-BOCS), assessing symptoms of OCD (5); the Repetitive Behavior Scale-Revised (RBS-R), assessing repetitive behaviors (6); and the Social Responsiveness Scale (SRS), assessing social communication skills (7).

## Whole-exome sequencing, alignment, variant calling, and quality control

Exome capture and sequencing were performed at the Yale Center for Genome Analysis (YCGA), using the NimbleGen SeqCap EZExomeV2 capture library (Roche NimbleGen, Madison, WI, USA) and the Illumina HiSeq 2500 platform (74 bp paired-end reads; Illumina, San Diego, CA, USA). We multiplexed six samples during each capture reaction and sequencing lane, pooling parents and probands when possible. Alignment and variant calling of the sequencing reads followed the latest Genome Analysis Toolkit (GATK) (8) Best Practices guidelines, as described previously (9). Reads were aligned using BWA-mem (10) to the b37 human reference sequence with decoy sequences. Picard's MarkDuplicates tool was used to mark PCR duplicates (https://broadinstitute.github.io/picard/). GATK was used to realign indels, recalibrate quality scores, and generate GVCF files for each sample using the HaplotypeCaller tool. All samples were called jointly using GATK's GenotypeGVCFs tool, variant score recalibration was applied to the called variants, and all variant call data was written to a VCF file. This pipeline uses GATK's Best Practices parameters and the default parameters for BWA and Picard. Only passing variants were used in downstream analyses. Variants were annotated using the RefSeq hg19 gene definitions and multiple external databases of variant population frequency, conservation scores, variation intolerance, mutation severity, and predicted functional effects using ANNOVAR (11).

Relatedness statistics were calculated based on the method of Manichaikul et al. (12), implemented in VCFtools v0.1.14.10 (13). Trios were omitted if expected family relationships were not confirmed or if there were unexpected relationships within or between families. Trios were omitted if > 5 de novo variants were observed. PLINK/SEQ (14) (i-stats; https://psychgen.u.hpc.mssm.edu/plinkseq/stats.shtml), PicardTools, and GATK DepthOfCoverage tools were used to generate quality metrics (Table S1). To identify outliers that might confound our case-control analysis, we performed principal components analysis (PCA) using this data. A scree plot determined the number of principal components accounting for the greatest proportion of variance, and we removed trios with family members falling more than three standard deviations from the mean in any of these principal components (Figure S1, Table S1). The R code PCA is provided below.

We used stringent thresholds for identifying de novo mutations because DNA from control subjects in the Simons Simplex Collection was not available for confirmation by Sanger sequencing. As previously described (9), de novo variants were called using an in-house script that required: (a) child is heterozygous for a variant, with alternate allele frequency between 0.3 and 0.7 in the child and < 0.05 in the parents; (b) sequencing depth (DP) ≥ 20 in all family members at the variant position; (c) alternate allele depth (AD) ≥ 5; (d) observed allele frequency (AC) < 0.01 (1%) among all cases and controls; and (e) mapping quality (MQ) ≥ 30. False positive calls were removed by in silico visualization. In our earlier whole-exome sequencing study of OCD using these same methods, Sanger sequencing yielded a 98.7% (147/149) confirmation rate (9). For this study, we performed Sanger sequencing in the proband and parents, confirming all (100%) de novo damaging variant calls (Mis-D and LGD) in pCMS trios. Sanger sequencing confirmation status for each variant is included in Table S2.

## Principal Component Analysis (PCA)

PCA was performed on all sequencing quality metrics (Table S1) in R using the following code:

library**(**xlsx**)**

library**(**"FactoMineR"**)**

library**(**"factoextra"**)**

library**(**"corrplot"**)**

## Load Data from Table S1, first tab

data1 **<-** read.delim**(**"Table_S1.xlsx"**)**

# select only certain columns

data1.temp **<-** data1**[**c**(**2,14**:**45**)]**

# make first column the row names

data1.active **<-** data.frame**(**data1.temp**[**,**-**1**]**, row.names**=**data1.temp**[**,1**])**

## Load additional data for later use (non-numeric labels/groups)

# select only certain columns

data2.temp **<-** data1**[**c**(**4,3,1**)]**

# make first column the row names

data2.active **<-** data.frame**(**data2.temp**[**,**-**1**]**, row.names**=**data2.temp**[**,1**])**

## Principal component analysis

pdf**(**"PCA_factor_maps.pdf"**)**

res.pca **<-** PCA**(**data1.active, scale.unit **=** **TRUE**, ncp **=** 10, graph **=** **TRUE**, axes **=** c**(**1,2**))**

dev.off**()**

print**(**res.pca**)**

## Export PCA coordinates to determine outliers (Table S1)

indcoord**<-**res.pca**$**ind**$**coord

write.xlsx**(**indcoord, "Table_S1.xlsx"**)**

## Estimate the number of components in Principal Component Analysis (Factominer)

sink**(**"EstmateNumberPCs.txt"**)**

estim_ncp**(**data1.active, ncp.min**=**0, ncp.max**=NULL**, scale**=TRUE**, method**=**"Smooth"**)**

sink**()**

## Variances of the principal components

eigenvalues **<-** res.pca**$**eig

## Make scree plot using base graphics : A scree plot is a graph of the eigenvalues/variances associated with components (Figure S1.A).

pdf**(**"ScreePlot.pdf"**)**

barplot**(**eigenvalues**[**, 2**]**, names.arg**=**1**:**nrow**(**eigenvalues**)**,

main **=** "Variances",

xlab **=** "Principal Components",

ylab **=** "Percentage of Variance",

col **=**"steelblue"**)**

lines**(**x **=** 1**:**nrow**(**eigenvalues**)**, eigenvalues**[**, 2**]**,

type**=**"b", pch**=**19, col **=** "red"**)**

dev.off**()**

## Make cumulative variance graph (Figure S1.B)

pdf**(**"ScreePlot_cumulative.pdf"**)**

barplot**(**eigenvalues**[**, 3**]**, names.arg**=**1**:**nrow**(**eigenvalues**)**,

main **=** "Variances",

xlab **=** "Principal Components",

ylab **=** "Cumulative Percentage of Variance",

col **=**"steelblue"**)**

lines**(**x **=** 1**:**nrow**(**eigenvalues**)**, eigenvalues**[**, 3**]**,

type**=**"b", pch**=**19, col **=** "red"**)**

dev.off**()**

## GRAPHS OF VARIABLES

pdf**(**"PCA_factor_maps_variables.pdf"**)**

fviz_pca_var**(**res.pca, col.var**=**"contrib"**)** **+** scale_color_gradient2**(**low**=**"white", mid**=**"blue", high**=**"red", midpoint**=**55**)+**theme_bw**()**

dev.off**()**

## GRAPHS OF INDIVIDUALS (Figure S1.C)

pca **=** prcomp**(**data1.active, scale **=** **TRUE)**

pdf**(**"PCA_prcomp_factor_map_indiv.pdf"**)**

plot**(**pca**$**x, pch **=** 20, col **=** c**(**rep**(**"red", 366**)**, rep**(**"blue", 1200**)))**

dev.off**()**

## Mutation rate calculations

Within each cohort, we calculated the rate of de novo mutations per base pair. For accurate rate calculation, we first determined the number of “callable” base pairs per family using the GATK DepthOfCoverage tool. We considered only bases covered at ≥ 20x in all family members, with base quality ≥ 20, and map quality ≥ 30; these thresholds match those required for GATK and de novo variant calling. For each cohort, we summed the “callable” base pairs in every family and used this number as the denominator for de novo rate calculations. Details for calculating these callable base pairs is below. The resulting rate was divided by two to give haploid rates. Confidence intervals were calculated using the *pois.conf.int (pois.exact)* function from the epitools v0.5-9 package in R. We compared de novo mutation rates in cases versus controls (burden analysis) using a one-tailed rate ratio test in R (https://cran.r-project.org/package=rateratio.test), considering only those variants present with a frequency of <0.001 in the ExAC v0.3.1 database (15).

## Calculating “callable” base pairs

The following command was used to calculate the callable base pairs in each trio:

java **-**jar GenomeAnalysisTK.jar -T DepthOfCoverage -R human_g1k_v37.fasta -o FamilyID **-**I FamilyID**.**list -L target_intersection.bed **--**minMappingQuality 30 **--**minBaseQuality 20 **--**summaryCoverageThreshold 20

FamilyID.list contains names and locations of the three trio bam files. The .bed file contains the genomic intervals over which to calculate the callable base pairs. To calculate the coding callable base pairs (used for coding mutation rates, e.g. synonymous, nonsynonymous, missense, etc., see Table 1, Table S2), we used a bed file with intervals spanning the intersection of both capture array target intervals and the RefSeq coding intervals (32,027,823 bp total). To calculate all callable base pairs (used for the total coding + noncoding mutation rate, see “All” in Table 1), we used a bed file with intervals spanning the intersection of both capture array target intervals (33,973,867 bp total). The number of coding and total callable base pairs for every family passing is listed in Table S1.

## Calculating expected mutation rates for downstream analyses

To perform subsequent maximum likelihood estimation (MLE) and TADA analyses, we used published per gene de novo mutation rates from unaffected parent-child trios (16). From the control samples in our dataset, we calculated the proportion of the overall coding mutation rate that comprised LGD and Mis-D mutations, and then used these proportions to calculate the expected LGD and Mis-D mutation rate per gene (Table S3).

The following R code was used to generate the expected mutation rates:

library**(**denovolyzeR**)**

library**(**plyr**)**

#######################

#### Mutation type fractions from controls in CMS project

#######################

# fractions of overall coding mutation rate for each variant type in SSC controls (see Table 1)

fracLGD **<-** 0.055747126

fracMisD **<-** 0.304597701

fracDamaging **<-** 0.360344828

#######################

#### Get published de novo mutation rtaes

#######################

denovolyzer **<-** viewProbabilityTable**()**

mutationProbs **<-** denovolyzer**[** , c**(**"geneName", "all"**)]**

mutationProbs **<-** rename**(**mutationProbs, c**(**"geneName"**=**"gene.name"**))** #rename column

mutationProbs **<-** rename**(**mutationProbs, c**(**"all"**=**"mut.rate"**))** #rename column

#save(mutationProbs, file = "denovolyzer_rates_all_unadjusted.RData")

#write.table(mutationProbs, "denovolyzer_rates_all_unadjusted.txt", sep="\t")

#######################

#### Add LGD and Mis-D, and Damaging de novo mutation rates based on fractions seen in our study

#######################

mutationProbs**$**lgd **<-** mutationProbs**$**mut.rate ***** fracLGD

mutationProbs**$**misD **<-** mutationProbs**$**mut.rate ***** fracMisD

mutationProbs$damaging <- mutationProbs$mut.rate * fracDamaging

save**(**mutationProbs, file **=** "de_novo_mutation_rates.RData"**)**

write.table**(**mutationProbs, "de_novo_mutation_rates.txt", sep**=**"\t"**)**

## TADA analysis

As shown in prior WES studies in neuropsychiatric disorders, a small number of rare de novo mutations in the same gene among unrelated individuals can provide considerable statistical power to establish association. To test this hypothesis in pCMS, we used the transmitted and de novo association (TADA-Denovo) test. TADA uses a Bayesian model that combines data from de novo mutations and population mutation rates to increase the power of gene discovery. While TADA has a version that can include inherited variants, we did not include inherited data in this study, because their confirmation rate is not known and their contribution to the TADA score is minimal, given their lower relative risks (17, 18). The code and documentation for this tool can be found here (http://wpicr.wpic.pitt.edu/WPICCompGen/TADA/TADA_homepage.htm).

We describe the parameters of this test in our prior WES studies of Tourette’s disorder and OCD (9, 19). The code and parameters used in the current study are given below. A low FDR-corrected q-value represents strong evidence for association. Genes with FDR q<0.3 are considered probable risk genes, and those with FDR q<0.1 are high-confidence risk genes.

source**(**"TADA.v1.1.R"**)**

# set.seed(100)

### read mutation rates and counts (see Table S3, second tab)

tada.file**=**"Table_S3.txt"

tada.data**=**read.table**(**tada.file,header**=**T**)**

### Number of mutations and TADA parameters

numLgdMutations **<-** **(**0.13*****118**)**

lgdRate **<-** numLgdMutations**/**118

numControlLgdMutations **<-** **(**0.066*****750**)**

controlLgdRate **<-** numControlLgdMutations**/**750

lgdRiskFraction **<-** **(**lgdRate **-** controlLgdRate**)** **/** lgdRate

numMis3Mutations **<-** **(**0.45*****118**)**

mis3Rate **<-** numMis3Mutations**/**118

numControlMis3Mutations **<-** **(**0.36*****750**)**

controlMis3Rate **<-** numControlMis3Mutations**/**750

mis3RiskFraction **<-** **(**mis3Rate **-** controlMis3Rate**)** **/** mis3Rate

numGenes **<-** 184 # from MLE analysis below

nPerms **<-** 1000

pi **<-** numGenes **/** nrow**(**tada.data**)**

pi0 **<-** 1**-**pi

numSilentMutations **<-** **(**0.32*****118**)**

numControlSilentMutations **<-** **(**0.32*****750**)**

dn.lof.lambda **<-** **(**numLgdMutations**)** **/** **(**numControlLgdMutations ***** **(**numSilentMutations**/**numControlSilentMutations**))**

dn.lof.relativeRisk **<-** 1 **+** **((**dn.lof.lambda**-**1**)** **/** pi**)**

dn.mis3.lambda **<-** **(**numMis3Mutations**)** **/** **(**numControlMis3Mutations ***** **(**numSilentMutations**/**numControlSilentMutations**))** # num Mis3 in ctrls, num silent in cases, num silent in ctrls

dn.mis3.relativeRisk **<-** 1 **+** **((**dn.mis3.lambda**-**1**)** **/** pi**)**

n.family **=** 118

n **=** data.frame**(**dn**=**n.family, ca**=NA**, cn**=NA)**

sample.counts **<-** list**(**cls1**=**n, cls2**=**n**)**

### create the mutational data used by TADA-Denovo

cls1.counts**=**data.frame**(**dn**=**tada.data**$**dn.cls1, ca**=NA**, cn**=NA)**

rownames**(**cls1.counts**)=**tada.data**$**gene.id

cls2.counts**=**data.frame**(**dn**=**tada.data**$**dn.cls2, ca**=NA**, cn**=NA)**

rownames**(**cls2.counts**)=**tada.data**$**gene.id

tada.counts**=**list**(**cls1**=**cls1.counts,cls2**=**cls2.counts**)**

### set up mutation rates

mu**=**data.frame**(**cls1**=**tada.data**$**mut.cls1,cls2**=**tada.data**$**mut.cls2**)**

### specify de novo only analyses

denovo.only**=**data.frame**(**cls1**=TRUE**,cls2**=TRUE)**

### set up parameters -

cls1**=** data.frame**(**gamma.mean.dn.**=**dn.lof.relativeRisk,beta.dn**=**1,gamma.mean.CC**=NA**,beta.CC**=NA** ,rho1**=NA**,nu1**=NA**,rho0**=NA**,nu0**=NA)**

cls2**=** data.frame**(**gamma.mean.dn**=**dn.mis3.relativeRisk,beta.dn**=**1,gamma.mean.CC**=NA**,beta.CC**=NA**,rho1**=NA**,nu1**=NA**,rho0**=NA**,nu0**=NA)**

hyperpar**=**list**(**cls1**=**cls1,cls2**=**cls2**)**

### running TADA-Denovo

re.TADA **<-** do.call**(**cbind.data.frame, TADA**(**tada.counts**=**tada.counts, sample.counts**=**sample.counts, mu**=**mu, hyperpar**=**hyperpar, denovo.only**=**denovo.only**))**

### Bayesian FDR control

re.TADA**$**qval**=**Bayesian.FDR**(**re.TADA**$**BF.total, pi0 **=** pi0**)**

### run permutation to get the null distributions to use for calculating p-values for TADA

re.TADA.null**=**do.call**(**cbind.data.frame, TADAnull**(**tada.counts**=**tada.counts, sample.counts**=**sample.counts, mu**=**mu, hyperpar**=**hyperpar, denovo.only**=**denovo.only, nrep**=**nPerms**))**

re.TADA**$**pval**=**bayesFactor.pvalue**(**re.TADA**$**BF.total,re.TADA.null**$**BFnull.total**)**

### display top 10 genes based on BF.total

re.TADA**[**order**(-**re.TADA**$**BF.total**)[**1**:**10**]**,**]**

### write all table to file - See Table S3

write.table**(**re.TADA, "TADA_denovo_Results.txt", sep**=**"\t"**)**

save.image**(**file**=**"TADA_denovo_Workspace.RData"**)**

## Maximum Likelihood Estimation (MLE) method for estimating the number of pCMS risk genes

We used a maximum likelihood estimation (MLE) method to estimate the number of genes contributing risk to pCMS, based on vulnerability to de novo damaging variants (20). For every number of risk genes from 1 to 2,500, we simulated 54 variants (the number of damaging de novo variants observed in probands in our case-control burden analysis). Variant simulations were performed 50,000 times at each number of risk genes. Following each simulation, a percentage of variants was randomly assigned to the risk genes. The percentage of variants assigned to risk genes was determined by the fraction of de novo damaging variants estimated to carry pCMS risk, and variant simulations were weighted by gene size and GC content (17). We then counted the number of risk and non-risk genes containing two variants and the number containing three or more variants. The frequency of concordance between our simulated and observed data was calculated. A curve was plotted to show the concordance frequency (y-axis) at each assumed number of risk genes (x-axis), and the peak was taken as the estimate of the most likely number of risk genes (20). See Figure S2.

The following R code was used to perform these calculations:

library**(**ggplot2**)**

library**(**parallel**)**

library**(**data.table**)**

plotDir **<-** getwd**()**

load**(**"de_novo_mutation_rates.RData"**)** # see Table S3, first tab

mutationProbs **<-** as.data.table**(**mutationProbs**)**

K **<-** 54 # total CMS de novo damaging mutations (Mis-D+LGD)

R2 **<-** 1 # number of above mutations hitting same gene twice

R3 **<-** 0 # number of above mutations hitting sane gene three times

M1 **<-** 54**/**118 # observed rate of de novo damaging mutations in CMS

M2 **<-** 225**/**750 # observed rate of de novo damaging mutations in controls

E **<-** **(**M1**-**M2**)/**M1 # estimating fraction of de novo damaging variants carrying risk

nPerms **<-** 50000 # number of permutations to perform at each assumed number of risk genes

maxGenes **<-** 2500 # perform permutations from 1 to this number

# get number of cores available

numCores **<-** max**(**1, detectCores**()** **-** 1**)**

#############################################################################

# FUNCTIONS

#############################################################################

getRecurrence **<-** **function(**G, mutationProbs, K, E, R2, R3, nPerms**){**

permutationVector **<-** lapply**(**1**:**nPerms, **function(**x**)** **{**

riskGeneIndex **<-** sample**(**1**:**nrow**(**mutationProbs**)**, G, replace **=** F**)**

riskGenes **<-** mutationProbs**[**riskGeneIndex,**]**

nonRiskGenes **<-** mutationProbs**[-**riskGeneIndex,**]**

C1 **<-** rbinom**(**1,K,E**)**

C2 **<-** K**-**C1

C1geneMutations **<-** sample**(**riskGenes**$**gene.name, C1, replace **=** T, prob **=** riskGenes**$**damaging**)**

C2geneMutations **<-** sample**(**nonRiskGenes**$**gene.name, C2, replace **=** T, prob **=** nonRiskGenes**$**damaging**)**

allGeneMutations **<-** c**(**C1geneMutations, C2geneMutations**)**

length**(**which**(**table**(**allGeneMutations**)==**2**))==**R2 **&** length**(**which**(**table**(**allGeneMutations**)>=**3**))==**R3

**})**

proportionMatchingObserved **<-** length**(**which**(**unlist**(**permutationVector**)))/**nPerms

**}**

#############################################################################

# RUN

#############################################################################

RNGkind**(**"L'Ecuyer-CMRG"**)**

set.seed**(**1**)**

mc.reset.stream**()**

permutationTest **<-** mclapply**(**1**:**maxGenes,

**function(**x**)** getRecurrence**(**x, mutationProbs, K, E, R2, R3, nPerms**)**,

mc.cores **=** numCores, mc.set.seed **=** T **)**

save**(**permutationTest, file **=** paste**(**"UpTo", maxGenes, "genes", nPerms, "perms", "MaxLikelihoodPermutation.RData", sep**=**"_"**))**

toPlot **<-** data.frame**(**likelihood **=** unlist**(**permutationTest**)**, nGenes **=** 1**:**length**(**unlist**(**permutationTest**)))**

save**(**toPlot, file **=** paste**(**"UpTo", maxGenes, "genes", nPerms, "perms", "MaxLikelihoodPermutationToPlot.RData", sep**=**"_"**))**

p **<-** ggplot**(**toPlot, aes**(**x**=**nGenes, y**=**likelihood**))**

p **<-** p **+** geom_line**()** **+** geom_smooth**()**

ggsave**(**file.path**(**plotDir, paste**(**"UpTo", maxGenes, "genes", nPerms, "perms", "MaxLikelihood.pdf", sep**=**"_"**)** **)**, p**)**

save.image**(**file **=** paste**(**"Workspace", nPerms, "perms.RData", sep**=**"_"**))**

## Predicting the number of pCMS risk genes identified by cohort size

Future discovery of risk genes was predicted as previously described (9, 19). Fixing the number of pCMS risk genes at 184 (from estimate above), we simulated de novo mutations, with the number of mutations matching the observed mutation rate in pCMS probands, and with mutation simulations weighted by gene size and GC content. Simulations were performed at each cohort size, from 25 to 3000, in increments of 25. Simulated variants were randomly assigned to the risk genes, with the percentage of variants assigned to risk genes determined by the fraction of de novo damaging variants estimated to carry pCMS risk. At each cohort size, 10,000 simulations were performed. LGD and Mis-D variants were simulated separately. Simulated variants were then combined and given as input to the TADA-Denovo algorithm, using the same parameters described above for the observed data. The number of high confidence (q<0.1) and probable (q<0.3) risk genes were recorded and plotted using polynomial regression fitting; this regression model allows prediction of the number of genes identified at a specified cohort size. See Figure S3.

The following R code was used to perform these calculations:

library**(**ggplot2**)**

library**(**parallel**)**

library**(**reshape2**)**

plotDir **<-** getwd**()**

source**(**file **=** "TADA.v1.1.R"**)**

load**(**"de_novo_mutation_rates.RData"**)** # use rates in Table S3

numLgdMutations **<-** **(**0.13*****118**)**

lgdRate **<-** numLgdMutations**/**118

numControlLgdMutations **<-** **(**0.066*****750**)**

controlLgdRate **<-** numControlLgdMutations**/**750

lgdRiskFraction **<-** **(**lgdRate **-** controlLgdRate**)** **/** lgdRate

numMis3Mutations **<-** **(**0.45*****118**)**

mis3Rate **<-** numMis3Mutations**/**118

numControlMis3Mutations **<-** **(**0.36*****750**)**

controlMis3Rate **<-** numControlMis3Mutations**/**750

mis3RiskFraction **<-** **(**mis3Rate **-** controlMis3Rate**)** **/** mis3Rate

numGenes **<-** 184

nPerms **<-** 10000

# get number of cores available

numCores **<-** max**(**1, detectCores**()** **-** 1**)**

pi **<-** 0.00937914

pi0 **<-** 1**-**pi

numSilentMutations **<-** **(**0.32*****118**)**

numControlSilentMutations **<-** **(**0.32*****750**)**

dn.lof.lambda **<-** **(**numLgdMutations**)** **/** **(**numControlLgdMutations ***** **(**numSilentMutations**/**numControlSilentMutations**))**

dn.lof.relativeRisk **<-** 1 **+** **((**dn.lof.lambda**-**1**)** **/** pi**)**

dn.mis3.lambda **<-** **(**numMis3Mutations**)** **/** **(**numControlMis3Mutations ***** **(**numSilentMutations**/**numControlSilentMutations**))** # num Mis3 in ctrls, num silent in cases, num silent in ctrls

dn.mis3.relativeRisk **<-** 1 **+** **((**dn.mis3.lambda**-**1**)** **/** pi**)**

############################################################################

# FUNCTIONS

############################################################################

getGenes **<-** **function(**numGenes_f**=**numGenes, mutationProbs_f, cohortSize_f, mutationRate_f, riskFraction_f, probability_f**=**c**(**"lgd", "mis3"**)[**1**]){**

numMutations **<-** ceiling**(**cohortSize_f ***** mutationRate_f**)**

riskGeneIndex **<-** sample**(**1**:**nrow**(**mutationProbs_f**)**, numGenes_f, replace **=** F**)**

riskGenes **<-** mutationProbs_f**[**riskGeneIndex,**]**

nonRiskGenes **<-** mutationProbs_f**[-**riskGeneIndex,**]**

C1 **<-** rbinom**(**1, numMutations, riskFraction_f**)**

C2 **<-** numMutations **-** C1

C1geneMutations **<-** sample**(**riskGenes**$**gene.name, C1, replace **=** T, prob **=** riskGenes**$**probability**)**

C2geneMutations **<-** sample**(**nonRiskGenes**$**gene.name, C2, replace **=** T, prob **=** nonRiskGenes**$**probability**)**

allGeneMutations **<-** c**(**C1geneMutations, C2geneMutations**)**

**}**

runIteration **<-** **function(**numGenes_f**=**numGenes, mutationProbs_f, cohortSize_f, lgdMutationRate_f, mis3MutationRate_f, lgdRiskFraction_f, mis3RiskFraction_f, nTadaRep_f **=** 100**){**

lgdMutations **<-** getGenes**(**numGenes, mutationProbs_f, cohortSize_f, lgdMutationRate_f, lgdRiskFraction_f, "lgd"**)**

lgdMutations_df **<-** data.frame**(**gene**=**lgdMutations, lof**=**1, mis3**=**0, stringsAsFactors **=** F**)**

mis3Mutations **<-** getGenes**(**numGenes, mutationProbs_f, cohortSize_f, mis3MutationRate_f, mis3RiskFraction_f, "lgd"**)**

mis3Mutations_df **<-** data.frame**(**gene**=**mis3Mutations, lof**=**0, mis3**=**1, stringsAsFactors **=** F**)**

combinedMutations **<-** rbind**(**lgdMutations_df, mis3Mutations_df**)**

combinedMutations **<-** aggregate**(**combinedMutations**[**,c**(**"lof", "mis3"**)]**, by**=**list**(**combinedMutations**$**gene**)**, sum**)**

colnames**(**combinedMutations**)** **<-** c**(**"gene.id", "dn.lof", "dn.mis3"**)**

tadaResults **<-** runTada**(**cohortSize_f **=** cohortSize_f, mutationTable_f **=** combinedMutations, mutationProbs_f **=** mutationProbs_f, nTadaRep_f **=** nTadaRep_f**)**

return**(**tadaResults**)**

**}**

runTada **<-** **function(**cohortSize_f, mutationTable_f, mutationProbs_f, nTadaRep_f **=** 100**){**

tada.data **<-** merge**(**mutationTable_f, mutationProbs_f**[**,c**(**"gene.name", "lgd", "mis3"**)]**, by.x**=**"gene.id", by.y**=**"gene.name"**)**

names**(**tada.data**)[**which**(**names**(**tada.data**)==**"lgd"**)]** **<-** "mut.lof"

names**(**tada.data**)[**which**(**names**(**tada.data**)==**"mis3"**)]** **<-** "mut.mis3"

n.family **=** cohortSize_f

n **=** data.frame**(**dn**=**n.family, ca**=NA**, cn**=NA)**

sample.counts **<-** list**(**cls1**=**n, cls2**=**n**)**

cls1.counts**=**data.frame**(**dn**=**tada.data**$**dn.lof, ca**=NA**, cn**=NA)**

rownames**(**cls1.counts**)=**tada.data**$**gene.id

cls2.counts**=**data.frame**(**dn**=**tada.data**$**dn.mis3, ca**=NA**, cn**=NA)**

rownames**(**cls2.counts**)=**tada.data**$**gene.id

tada.counts**=**list**(**cls1**=**cls1.counts,cls2**=**cls2.counts**)**

mu**=**data.frame**(**cls1**=**tada.data**$**mut.lof,cls2**=**tada.data**$**mut.mis3**)**

denovo.only**=**data.frame**(**cls1**=TRUE**,cls2**=TRUE)**

cls1**=** data.frame**(**gamma.mean.dn**=**dn.lof.relativeRisk,beta.dn**=**1,gamma.mean.CC**=NA**,beta.CC**=NA** ,rho1**=NA**,nu1**=NA**,rho0**=NA**,nu0**=NA)**

cls2**=** data.frame**(**gamma.mean.dn**=** dn.mis3.relativeRisk,beta.dn**=**1,gamma.mean.CC**=NA**,beta.CC**=NA**,rho1**=NA**,nu1**=NA**,rho0**=NA**,nu0**=NA)**

hyperpar**=**list**(**cls1**=**cls1,cls2**=**cls2**)**

re.TADA **<-** do.call**(**cbind.data.frame, TADA**(**tada.counts**=**tada.counts, sample.counts**=**sample.counts, mu**=**mu, hyperpar**=**hyperpar, denovo.only**=**denovo.only**))**

re.TADA**$**qval**=**Bayesian.FDR**(**re.TADA**$**BF.total, pi0 **=** pi0**)**

tadaResults **<-** re.TADA**[**order**(**re.TADA**$**qval, decreasing **=** F**)**, **]**

probableGenes **<-** length**(**which**(**tadaResults**$**qval**<**0.3**))**

highConfidenceGenes **<-** length**(**which**(**tadaResults**$**qval**<**0.1**))**

return**(**data.frame**(**probable **=** probableGenes, highConfidence **=** highConfidenceGenes**))**

**}**

############################################################################

# RUN

############################################################################

RNGkind**(**"L'Ecuyer-CMRG"**)**

set.seed**(**1**)**

mc.reset.stream**()**

tadaSimulations **<-** mclapply**(**seq**(**from**=**25, to**=**3000, by**=**25**)**, **function(**x**)**

lapply**(**1**:**nPerms, **function(**y**)** runIteration**(**numGenes_f **=** numGenes, mutationProbs_f **=** mutationProbs, cohortSize_f **=** x, lgdMutationRate_f **=** lgdRate, mis3MutationRate_f **=** mis3Rate, lgdRiskFraction_f **=** lgdRiskFraction, mis3RiskFraction_f **=** mis3RiskFraction**))**,

mc.cores **=** numCores, mc.set.seed **=** T**)**

save**(**tadaSimulations, file **=** paste**(**"tadaSimulations", nPerms, "perms", "forGeneDiscoveryEstimate_noPval.RData", sep**=**"_"**))**

resultsByCohortSize **<-** lapply**(**tadaSimulations, **function(**x**)** do.call**(**rbind, x**))**

averageGeneDiscoveryByCohortSize **<-** lapply**(**resultsByCohortSize, **function(**x**)** apply**(**x, 2, mean**))**

averageGeneDiscoveryByCohortSize_DF **<-** as.data.frame**(**do.call**(**"rbind", averageGeneDiscoveryByCohortSize**))**

averageGeneDiscoveryByCohortSize_DF**$**cohortSize **<-** seq**(**from**=**25, to**=**3000, by**=**25**)**

save**(**averageGeneDiscoveryByCohortSize_DF, file **=** paste**(**"averageGeneDiscoveryByCohortSize", nPerms, "perms", ".RData", sep**=**"_"**))**

toPlot **<-** melt**(**averageGeneDiscoveryByCohortSize_DF, measure.vars**=**c**(**"probable", "highConfidence"**)**,

variable.name **=** "confidenceThreshold", value.name **=** "numGenes"**)**

save**(**toPlot, file **=** paste**(**"averageGeneDiscoveryByCohortSizetoPlot", nPerms, "perms", ".RData", sep**=**"_"**))**

p **<-** ggplot**(**toPlot, aes**(**x**=**cohortSize, y**=**numGenes, col**=**confidenceThreshold**))**

p **<-** p **+** geom_line**()**

ggsave**(**p, file**=**file.path**(**plotDir, paste**(**"averageGeneDiscoveryByCohortSize", nPerms, "perms.pdf", sep**=**"_"**)))**

## Gene set overlap

We used DNENRICH (14) (https://statgen.bitbucket.io/dnenrich/index.html) to test whether pCMS genes found to have de novo damaging mutations in our study (52 genes after excluding two genes with de novo damaging variants in controls) were significantly enriched among previously reported genes in autism (ASD), schizophrenia (SCZ), developmental disorders (DD), Tourette’s disorder (TD), obsessive-compulsive disorder (OCD), intellectual disability (ID), and epileptic encephalopathy (EE). Gene lists for ASD, SCZ, DD, ID, and EE were obtained from Gene4Denovo, an integrated database for de novo mutations in humans (21), version 07-08-2022 (http://www.genemed.tech/gene4denovo/home). TD (22) and OCD (9, 23) genes were obtained from recently published WES studies. For the TD gene list, we removed variants reported in subjects with comorbid OCD, as this phenotype information was readily available. Additional ASD gene lists were obtained from the SFARI Gene online database (https://gene.sfari.org/about-gene-scoring/criteria/), version 10-28-2022. ASD genes in this database have been stratified into four categories, based on manually curated strength of evidence from human genetics studies. Finally, we curated lists of genes harboring damaging de novo mutations in ASD probands from the Simons Simplex Collection (SSC) for whom stereotyped behavior scores (Stereotyped Behavior Score from the RBS-R, Repetitive Behavior Scale-Revised) were available. De novo mutation data from SSC probands was obtained from denovo-db (http://denovo-db.gs.washington.edu/, version 1.6.1). RBS-R Stereotyped Behavior Score data was obtained from the Simons Foundation. Because we were particularly interested in the question of whether our pCMS cohort share genes harboring de novo damaging mutations with SSC probands having high stereotypy scores, we assembled gene lists from SSC subjects with stereotypy scores in the 90^th^ percentile (high stereotypies) and those in the 10^th^ percentile (low stereotypies). Gene lists are provided in Table S4, first tab.

DNENRICH simulates random mutations while accounting for gene size, trinucleotide context, and mutational effect. We performed 100,000 permutations, comparing the observed and expected overlap with each gene set. In both the mutation file (mutations_ocd_damaging.mut) and the gene set lists file (gene_lists.set), we followed the suggested use of “gene-weighting” that has been used in prior studies to assess overlap of genes harboring de novo mutations (14). We assigned gene weights such that a gene hit recurrently by independent de novo mutations has a weight equal to the number de novo mutations. Empirical p-values were generated, based on a one-sided enrichment analysis under a binomial model of greater than expected hits per gene set. We tested for overlap between our pCMS genes and those in each of the mentioned gene lists. Results are provided in Table S4, third tab.

The following Linux commands were used to run the DNENRICH analysis:

dnenrich **.** 100000 alias.txt refseq_gene_sizes.txt gene_lists.set mutations_ocd_damaging.mut **>** results**;**

csh extractDnenrichResults.csh results **>** results.txt

## Exploratory gene ontology, network, and spatiotemporal analyses

To explore whether genes harboring de novo damaging variants in our pCMS probands (52 genes after excluding two genes, *ADGRB3* and *EIF4ENIF1,* with de novo damaging variants also found in SSC controls, Table 2, S2 Table) are enriched for gene ontology terms relating to biological processes, cellular components, and molecular function, we used ConsensusPathDB (24) (http://cpdb.molgen.mpg.de/, Release 35 [05.06.2021], accessed 12/28/2022). This tool integrates human protein and genetic interaction networks from 32 databases and interactions curated from the literature. The following default settings were used for ConsensusPathDB: gene set analysis 🡪 over-representation analysis; gene identifier type: gene symbol (HGNC symbol); Gene ontology categories: gene ontology level 2 categories, select all (biological processes, molecular function, cellular component), p-value cutoff = 0.05. Results are in Table S5, second tab.

We performed a similar exploratory gene ontology analysis using a second complementary tool, Enrichr (<https://maayanlab.cloud/Enrichr/>, 2021 Release). The following analyses modules were used: GO Biological Process 2021, GO Molecular Function 2021, GO Cellular Component 2021. Results are in Table S5, first tab.

We used Ingenuity Pathway Analysis (IPA, content version 81348237, release date 2022-09-15; Ingenuity Systems, <http://www.ingenuity.com/>) to identify the most significant gene networks based on our list of 52 pCMS genes. The networks are ranked by a score that reflects the likelihood of the genes in the network being associated with each other due to chance, with highest scores assigned to those with the lowest likelihood of being due to chance. The following default settings were used for IPA: Reference set: Ingenuity Knowledge Base (Genes Only); direct and indirect relationships; consider only relationships where species = human and confidence = experimentally observed. See Table S6.

For spatiotemporal enrichment analysis, we used our same list of 52 genes (Table 2, S2 Table) and asked whether these genes have known expression patterns that cluster within certain anatomical brain regions or within certain developmental time periods. To perform this analysis, we used data from the Brainspan Atlas of the Developing Human Brain (25) as implemented in the Specific Enrichment Analysis (SEA) tool (http://genetics.wustl.edu/jdlab/csea-tool-2/, version 1.1, accessed 10/20/2022). For this analysis, we used a specificity index threshold (pSI) of 0.05 (26). Results are in Table S5, fourth tab. Fisher’s Exact p-values are uncorrected for multiple comparisons.

**References**

1. J. M. Miller, H. S. Singer, D. D. Bridges, H. R. Waranch, Behavioral therapy for treatment of stereotypic movements in nonautistic children. *Journal of child neurology* **21**, 119-125 (2006).

2. S. Ehlers, C. Gillberg, L. Wing, A screening questionnaire for Asperger syndrome and other high-functioning autism spectrum disorders in school age children. *J Autism Dev Disord* **29**, 129-141 (1999).

3. J. S. March, J. D. Parker, K. Sullivan, P. Stallings, C. K. Conners, The Multidimensional Anxiety Scale for Children (MASC): factor structure, reliability, and validity. *J Am Acad Child Adolesc Psychiatry* **36**, 554-565 (1997).

4. G. J. DuPaul, T. J. Power, A. D. Anastopoulos, R. Reid, *ADHD Rating Scale—IV: Checklists, norms, and clinical interpretation* (Guilford Press, 1998).

5. L. Scahill *et al.*, Children's Yale-Brown Obsessive Compulsive Scale: reliability and validity. *J Am Acad Child Adolesc Psychiatry* **36**, 844-852 (1997).

6. J. W. Bodfish, F. J. Symons, D. E. Parker, M. H. Lewis, Varieties of repetitive behavior in autism: comparisons to mental retardation. *J Autism Dev Disord* **30**, 237-243 (2000).

7. J. N. Constantino (2005) Social Responsiveness Scale (SRS). (Western Psychological Services, Los Angeles, CA).

8. A. McKenna *et al.*, The Genome Analysis Toolkit: a MapReduce framework for analyzing next-generation DNA sequencing data. *Genome research* **20**, 1297-1303 (2010).

9. C. Cappi *et al.*, De Novo Damaging DNA Coding Mutations Are Associated With Obsessive-Compulsive Disorder and Overlap With Tourette's Disorder and Autism. *Biol Psychiatry* **87**, 1035-1044 (2020).

10. H. Li, R. Durbin, Fast and accurate long-read alignment with Burrows-Wheeler transform. *Bioinformatics* **26**, 589-595 (2010).

11. K. Wang, M. Li, H. Hakonarson, ANNOVAR: functional annotation of genetic variants from high-throughput sequencing data. *Nucleic acids research* **38**, e164 (2010).

12. A. Manichaikul *et al.*, Robust relationship inference in genome-wide association studies. *Bioinformatics* **26**, 2867-2873 (2010).

13. P. Danecek *et al.*, The variant call format and VCFtools. *Bioinformatics* **27**, 2156-2158 (2011).

14. M. Fromer *et al.*, De novo mutations in schizophrenia implicate synaptic networks. *Nature* **506**, 179-184 (2014).

15. M. Lek *et al.*, Analysis of protein-coding genetic variation in 60,706 humans. *Nature* **536**, 285-291 (2016).

16. J. S. Ware, K. E. Samocha, J. Homsy, M. J. Daly, Interpreting de novo Variation in Human Disease Using denovolyzeR. *Current protocols in human genetics / editorial board, Jonathan L. Haines ... [et al.]* **87**, 7.25.21-15 (2015).

17. X. He *et al.*, Integrated model of de novo and inherited genetic variants yields greater power to identify risk genes. *PLoS Genet* **9**, e1003671 (2013).

18. S. J. Sanders *et al.*, Insights into Autism Spectrum Disorder Genomic Architecture and Biology from 71 Risk Loci. *Neuron* **87**, 1215-1233 (2015).

19. A. J. Willsey *et al.*, De Novo Coding Variants Are Strongly Associated with Tourette Disorder. *Neuron* **94**, 486-499 e489 (2017).

20. J. Homsy *et al.*, De novo mutations in congenital heart disease with neurodevelopmental and other congenital anomalies. *Science (New York, N.Y.)* **350**, 1262-1266 (2015).

21. G. Zhao *et al.*, Gene4Denovo: an integrated database and analytic platform for de novo mutations in humans. *Nucleic acids research* **48**, D913-d926 (2020).

22. S. Wang *et al.*, De Novo Sequence and Copy Number Variants Are Strongly Associated with Tourette Disorder and Implicate Cell Polarity in Pathogenesis. *Cell reports* **24**, 3441-3454.e3412 (2018).

23. M. Halvorsen *et al.*, Exome sequencing in obsessive-compulsive disorder reveals a burden of rare damaging coding variants. *Nat Neurosci* **24**, 1071-1076 (2021).

24. R. Herwig, C. Hardt, M. Lienhard, A. Kamburov, Analyzing and interpreting genome data at the network level with ConsensusPathDB. *Nature protocols* **11**, 1889-1907 (2016).

25. H. J. Kang *et al.*, Spatio-temporal transcriptome of the human brain. *Nature* **478**, 483-489 (2011).

26. J. D. Dougherty, E. F. Schmidt, M. Nakajima, N. Heintz, Analytical approaches to RNA profiling data for the identification of genes enriched in specific cells. *Nucleic acids research* **38**, 4218-4230 (2010).
