# Supplementary material for: Primary complex motor stereotypies are associated with de novo damaging DNA coding mutations that identify KDM5B as a risk gene
Source: PLoS One. 2023 Oct 3;18(10):e0291978. doi: 10.1371/journal.pone.0291978 (PMC10547198; doi:10.1371/journal.pone.0291978)
Supplement: Attachment — Submitted filename: Response to Reviewers of PONE-D-21-39338.docx [file pone.0291978.s013.docx]

April 11, 2023

Emily Chenette, Ph.D.
Editor-in-Chief, *PLOS One*

Dear Dr. Chenette:

Thank you for your interest in a revised version of our manuscript entitled "Primary complex motor stereotypies are associated with de novo damaging DNA coding mutations that identify KDM5B as a risk gene". We thank the Reviewers for their helpful comments, and we address each of their concerns in detail below.

**Reviewer 1:**

We appreciate the reviewer’s comment that our data presentation is very complete and comprehensive, and our data analysis is described in detail.

***Comment 1.1: KDM5B is involved in autosomal recessive Intellectual Disability (OMIM 605393). This is not discussed at all in the manuscript. Did/Do the parents of children with AR KDM5B mutations display any evidence of CMS? The fact that KDM5B is a known Intellectual Disability gene needs to be clear in this study.***

***Response 1.1:*** We have updated our discussion about *KDM5B* to cite reports of other disease associations, including intellectual disability (ID) and developmental disorders (DD) more broadly. We clarify that while dominant disease variants may exist, these ID/DD cases show an autosomal recessive inheritance pattern. For example, ID cases reported in the literature with *KDM5B* mutations harbor inherited homozygous or compound heterozygous mutations in this gene. This contrasts with our CMS probands’ de novo heterozygous mutations. It has not been reported whether parents of children with recessive *KDM5B* mutations display evidence of CMS. It is certainly interesting to see how different mutations and inheritance patterns in this gene can lead to a spectrum of phenotypic outcomes, including ASD, ID/DD, congenital heart disease, and now CMS. We feel our study adds justification for studying the pleiotropic developmental consequences of these various mutations in *KDM5B*.

***Comment 1.2: It is also concerning that KDM5B did not show up in the DNENRICH analysis, raising questions about the validity of the DNENRICH data. How can we be confident that the DNENRICH dataset is correct and complete?***

***Response 1.2:*** Our DNENRICH analysis assesses the degree of overlap among de novo damaging mutations found in our CMS probands and de novo damaging mutations reported in other neuropsychiatric conditions. As discussed in Response 1.1, de novo damaging mutations in *KDM5B* have not been reported in intellectual disability, so this gene does not show up in our DNENRICH analysis. We focus on de novo variants in this study, as they have proven powerful for risk gene discovery in multiple neuropsychiatric disorders, including developmental delay and intellectual disability, given their low background rate in the population. Throughout the text, we now clarify that our DNENRICH analysis targets gene lists of de novo damaging variants.

***Comment 1.3: For the DNENRICH analysis, you are looking at overlapping gene sets. Why, then, does KDM5B get counted twice? Even though it is identified in two CMS patients, it is still just one gene and should be treated as so for gene overlap enrichment analysis. It is possible that counting KDM5B as one gene will reduce the statistical significance of these overlapping gene sets.***

***Response 1.3:*** Thank you for alerting us to this potential bias in our analysis. In our revised manuscript, we adjusted our DNENRICH analyses of overlapping gene sets to account for lists where independent de novo mutations are found in the same gene in more than one individual. In this scenario, we no longer make duplicate gene entries. Instead, we now follow recommendations by the authors of DNENRICH and its use in prior studies. We now include each gene only one time per list, and we assign gene weights such that a gene harboring independent de novo mutations has a weight equal to the number of de novo mutations. Importantly, we made this adjustment for our CMS gene list and for the candidate gene sets, which we now obtain from Gene4Denovo. In this way, we are now comparing our CMS de novo gene list to candidate de novo gene sets (e.g., Tourette’s, OCD, autism, ID), and each list/set is now weighted using the same method and analyzed using a tool specifically designed to determine the statistical significance of overlap between lists of de novo mutations. These changes are detailed in the Supplementary Methods. By using DNENRICH more robustly, we continue to find statistical significance for overlap between our pCMS genes, and gene sets for Tourette’s disorder, autism spectrum disorder (ASD), and ASD with high stereotypy scores.

***Comment 1.4: The authors discuss 52 genes with de novo loss of function variants in the CMS cohort. This list of genes and mutations is not included in the manuscript. It would be useful if this list were included in a separate table. I tried to reproduce this list by selecting LDG and Mis-D variants from Table S2 and could not.***

***Response 1.4:*** Thank you for this suggestion. We now include the list of 52 variants in Table S2. Additionally, we now include Table 2 in the main manuscript, providing a list of 52 genes with de novo loss of function variants in the CMS cohort.

***Comment 1.5: The Gene Ontology analysis is done using http://cpdb.molgen.mpg.de/ release from 2019. This should be redone with the 2021 release of the program or using a different program with an up to date ontology.***

***Response 1.5:*** As suggested by Reviewer 1, we repeated our exploratory gene ontology analysis using the latest release of ConsensusPathDB. In addition, as suggested by Reviewer 2, we added a second tool for gene ontology analysis using the latest version of Enrichr (see Response 2.3, below).

***Comment 1.6: The title and the conclusions are overstated and misleading. The title refers to risk "genes" however, only one risk gene was identified (KDM5B), and that gene is confounded by the fact that it is a known autism risk gene and is involved in autosomal recessive ID. The title and abstract also refer to the identification of candidate biological pathways involved in CMS. This is a big overstatement. No biological pathway is identified for CMS in this study. The GO enrichment is minimal, with very few genes contributing to the enriched terms. This is not enough evidence to [say] that Biological pathways have been identified. Demethylation is not a biological pathway; it is an enzymatic function that may be linked to many biological pathways.***

***Response 1.6:*** We agree with Reviewer 1 that our title and conclusions could be interpreted as overreaching, and we inadvertently conflated biological pathways and gene ontology terms in our title, results, and discussion. In this revision, we made several changes to clarify our results and the significance of our findings: (1) The title of our report now clearly states that we identified only one risk gene -- *KDM5B*; (2) We removed wording from our title and abstract that overstated the significance of our gene ontology analyses, and we now refer to these analyses as exploratory; (3) We no longer refer to the identification of “biological pathways.” Instead, we refer to these analyses more accurately as tests for enrichment of gene ontology terms.

**Reviewer 2:**

We appreciate the reviewer’s comment that our analysis is well-described.

***Comment 2.1: No sequencing data are available.***

***Response 2.1:*** We now provide raw variant call files for all subjects included in our study. These data have been submitted to the Dryad Digital Repository and assigned a unique DOI (doi:10.5061/dryad.rfj6q57d5). The data submission is currently in “private for peer review” status, so this DOI will not be live until the manuscript is accepted for publication. However, a private URL to this data is provided by Dryad for use during peer review: https://datadryad.org/stash/share/HU8cwTlay7QNbwTWYhuuBiZcSeV75dtgmkzsB4B08N0

Clicking this link immediately launches a download of the data files in the repository.

***Comment 2.2: Sanger sequencing validation was not performed.***

***Response 2.2:*** Since our initial submission, we performed Sanger sequencing on variants in our CMS probands and parents, confirming all de novo damaging variant calls included in our analyses. This is now explained in the Supporting Information. We also added the results of Sanger sequencing variant confirmations to Table S2 (second tab, column AD).

***Comment 2.3: Gene Ontology analysis was performed only with a single unique approach.***

***Response 2.3:*** We now add a second tool for exploratory gene ontology analysis using the latest version of Enrichr (https://maayanlab.cloud/Enrichr/). We highlight those results that are shared between tools.

Thank you for the opportunity to improve this manuscript.

Sincerely,

Thomas V. Fernandez, M.D.

Associate Professor, Yale Child Study Center & Psychiatry

Vice Chair for Research, Yale Child Study Center

Co-Director, Tic Disorder/OCD/ADHD Clinic
