# Supplementary material for: Primary complex motor stereotypies are associated with de novo damaging DNA coding mutations that identify KDM5B as a risk gene
Source: PLoS One. 2023 Oct 3;18(10):e0291978. doi: 10.1371/journal.pone.0291978 (PMC10547198; doi:10.1371/journal.pone.0291978)
Supplement: Attachment — Submitted filename: Response to Reviewers of PONE-D-21-39338 - July 2023.docx [file pone.0291978.s014.docx]

August 2, 2023

Emily Chenette, Ph.D.
Editor-in-Chief, *PLOS One*

Dear Dr. Chenette:

Thank you for the opportunity to submit a revised version of our manuscript, "Primary complex motor stereotypies are associated with *de novo* damaging DNA coding mutations that identify *KDM5B* as a risk gene" (PONE-D-23-11138). We thank the Reviewers and Academic Editor for their helpful comments, and we address each of their points below.

**Academic Editor:**

***Comment 1:* *Please ensure that your manuscript meets PLOS ONE's style requirements, including those for file naming.***

**Response 1:** We reviewed the PLOS ONE style templates and made a few minor formatting changes in the title page, main body, and supporting information text. These changes are marked in the ‘Track Changes’ versions of these files. We also made changes to several file names, following the style requirements.

***Comment 2:* *Please provide an amended statement that declares *all* the funding or sources of support (whether external or internal to your organization) received during this study, as detailed online in our guide for authors. Please also include the statement “There was no additional external funding received for this study.” in your updated Funding Statement.***

**Response 2:** In the cover letter, we confirmed that there are no changes to the financial disclosure for this manuscript, and no additional external funding was received for this study. As instructed, an amended Funding Statement was included in the cover letter.

***Comment 3:* *Please confirm that [the competing interests statement] does not alter your adherence to all PLOS ONE policies on sharing data and materials, by including the following statement: "This does not alter our adherence to PLOS ONE policies on sharing data and materials.” If there are restrictions on sharing of data and/or materials, please state these.***

**Response 3:** In the cover letter, we confirmed that our stated competing interests (unchanged) do not alter our adherence to PLOS ONE policies on sharing data and materials. As instructed, an amended Competing Interests Statement was included in the cover letter.

***Comment 4:* *We note that you have stated that you will provide repository information for your data at acceptance. Should your manuscript be accepted for publication, we will hold it until you provide the relevant accession numbers or DOIs necessary to access your data. If you wish to make changes to your Data Availability statement, please describe these changes in your cover letter, and we will update your Data Availability statement to reflect the information you provide.***

**Response 4:** We have not requested any changes to our Data Availability Statement. Our data have been submitted to the Dryad Digital Repository and have already been assigned a unique DOI (doi:10.5061/dryad.rfj6q57d5). The data submission is currently in “private for peer review” status, which means it is not yet available publicly. However, a private URL to this data is provided by Dryad for use during peer review: https://datadryad.org/stash/share/HU8cwTlay7QNbwTWYhuuBiZcSeV75dtgmkzsB4B08N0

We intend to change the data submission to allow public access when our manuscript is accepted for publication.

***Comment 5:* *Please review your reference list to ensure that it is complete and correct. If you have cited papers that have been retracted, please include the rationale for doing so in the manuscript text, or remove these references and replace them with relevant current references. Any changes to the reference list should be mentioned in the rebuttal letter that accompanies your revised manuscript. If you need to cite a retracted article, indicate the article’s retracted status in the References list and also include a citation and full reference for the retraction notice.***

**Response 5:** We reviewed our reference list and made the following changes to reflect pre-print articles that are now published in peer reviewed journals:

| *Old reference* | *New reference* |
| --- | --- |
| Cappi C, Oliphant ME, Peter Z, Zai G, Sullivan CA, Gupta AR, et al. De novo damaging coding mutations are strongly associated with obsessive-compulsive disorder and overlap with autism. bioRxiv. 2017:127712. | Cappi C, Oliphant ME, Peter Z, Zai G, Conceicao do Rosario M, Sullivan CAW, et al. De Novo Damaging DNA Coding Mutations Are Associated With Obsessive-Compulsive Disorder and Overlap With Tourette's Disorder and Autism. Biol Psychiatry. 2020;87(12):1035-44. |
| Satterstrom FK, Kosmicki JA, Wang J, Breen MS, De Rubeis S, An J-Y, et al. Large-scale exome sequencing study implicates both developmental and functional changes in the neurobiology of autism. bioRxiv. 2019:484113. | Satterstrom FK, Kosmicki JA, Wang J, Breen MS, De Rubeis S, An JY, et al. Large-Scale Exome Sequencing Study Implicates Both Developmental and Functional Changes in the Neurobiology of Autism. Cell. 2020;180(3):568-84 e23. |

Several additional references have been added to address points raised by reviewers. These are mentioned in our responses to Reviewers 1 and 2, and also listed here for convenience:

Olfson E, Lebowitz ER, Hommel G, Pashankar N, Silverman WK, Fernandez TV. Whole-exome DNA sequencing in childhood anxiety disorders identifies rare de novo damaging coding variants. Depress Anxiety. 2022;39(6):474-84.

Chen CY, Tian R, Ge T, Lam M, Sanchez-Andrade G, Singh T, et al. The impact of rare protein coding genetic variation on adult cognitive function. Nat Genet. 2023;55(6):927-38.

Huang Y, Bodnar D, Chen CY, Sanchez-Andrade G, Sanderson M, Shi J, et al. Rare genetic variants impact muscle strength. Nat Commun. 2023;14(1):3449.

Deciphering Developmental Disorders Study. Prevalence and architecture of de novo mutations in developmental disorders. Nature. 2017;542(7642):433-8.

We confirmed that no retracted papers have been cited.

**Reviewer 1:**

We appreciate the reviewer’s compliments about our study and agreement with our identification of a likely candidate risk gene.

***Comment 1.1: The only potential problem with the conclusions is the fact that the controls were genotyped separately as part of another study. The authors could add a paragraph explaining why this is not a problem and perhaps adding some data on sequencing depth and other features that could be different between the methods. But it is standard to combine large datasets of sequenced sampled from different labs, so I presume this was all done correctly.***

***Response 1.1:*** We agree that separate sequencing sets may introduce potential confounding variables and batch effects. We were sensitive to this possibility when designing our study and took precautions to guard against this potential bias in our data, consistent with common practices in whole-exome sequencing studies. To explain this more clearly, we added the following text to the Methods section (Mutation rate and gene recurrence) of the main manuscript. Furthermore, sequencing metrics are included in S1 Table.

“Because our cases and controls were sequenced at different times, we took precautions to ensure that batch effects, including differences in sequencing depth and quality, did not influence our comparisons. First, we compared cases and controls that were sequenced on the same sequencing platform and using the same capture library. Second, we considered only “callable” bases, defined as loci with ≥ 20x sequencing depth in all family members, with base quality ≥ 20, and map quality ≥ 30; these thresholds match those required for GATK and de novo variant calling. Third, for each cohort, we summed the “callable” base pairs in every family and used this number as the denominator for de novo rate calculations. In this way, we normalized the de novo rates to guard against any residual differences in sequencing depth or quality, and we compared these normalized rates between cases and controls. This method of comparing different batches of sequencing data has been used in several prior studies (e.g., Willsey et al., 2017; Wang et al., 2018; Cappi et al., 2020; Olfson et al., 2022).”

**Reviewer 2:**

We appreciate the reviewer’s assessment that our findings represent a useful addition to the literature related to the gene *KDM5B*.

***Comment 2.1: My main additional comment is that the authors’ response to Reviewer 1 no longer holds up. Specifically, a recent paper by Chen et al. (Nature Genetics 2023 https://doi.org/10.1038/s41588-023-01398-8) establishes KDM5B as a dosage-sensitive gene for which heterozygote carriers show attenuated symptoms of phenotypes observed in individuals with autosomal-recessive KDM5B intellectual disability (ID) syndrome. Additionally, a more recent paper by Huang et al. (NCOMMS 2023; https://www.nature.com/articles/s41467-023-39247-1) introduces heterozygote variants in KDM5B as relevant for hand-grip strength. Importantly, Chen et al. also report that KDM5B has a fairly high de novo mutation rate, which sets it apart from the majority of other genes linked to ASD and ID. Notably, unlike the authors’ paper Chen et al. did not link KDM5B to pCMS (which does not seem to be well-curated in UK Biobank). They also followed a population rather than a trio-sequencing approach, which only indirectly allows for assumptions on whether variants are truly de novo. From this perspective, the current paper would certainly add to the literature if the authors assumptions for TADA-Denovo analyses (which I am not familiar with) and significance cut-offs for KDM5B remain unchanged in the light of variant frequency and pLi information from UK Biobank exome sequencing results.***

***Response 2.1:*** Thank you for bringing these studies by Chen et al. (2023) and Huang et al. (2023) to our attention. Based on the findings from these studies, which were both published after our last submission, we modified text in the Discussion section regarding the pleiotropic effects of mutations in this gene, copied here. Our variant frequency threshold of 0.1% agrees with thresholds used in these studies, and results from TADA do not depend on pLI data.

“More recently, this gene has also been implicated in congenital heart disease risk, embryonic development, and DNA repair, adult cognitive function, and muscle strength (65-70). KDM5B has been identified as high-confidence risk gene in ASD via the detection of heterozygous de novo damaging variants in WES studies (48, 56) and for developmental disorders more broadly (71). While dominant disease variants may exist, individuals reported in the literature with intellectual disability/developmental delay harboring KDM5B mutations often show an autosomal recessive inheritance pattern, including inherited homozygous or compound heterozygous mutations in this gene (72, 73), while heterozygous mutations occur more frequently in probands from the Deciphering Developmental Disorders Study (74). A recent study by Chen et al. (69) identified KDM5B as one of eight genes associated with adult cognitive function through rare protein-truncating and damaging missense variants. Consistent with prior reports, they identified a gene dose effect, whereby individuals with rare heterozygous protein truncating variants showed higher adult cognitive function measurements compared to those with homozygous damaging mutations (69). Both this study and another recent report by Huang et al. (70) found a significant association between rare variants in KDM5B and hand grip strength, a phenotype related to muscle function, and one of several additional phenotypes found to be associated with KDM5B (69). Interestingly, KDM5B has a relatively high rate of protein-truncating variants, with a rate of approximately 1 in 1,900 subjects in the UK Biobank sample. This is in contrast to most other genes linked to neurodevelopmental phenotypes. Considering the substantial pleiotropic and gene dosage effects reported in several studies to date, This contrasts with our CMS probands’ de novo heterozygous mutations. It is interesting to see how different mutations and inheritance patterns in this gene can lead to a spectrum of phenotypic outcomes, including ASD, ID/DD, congenital heart disease, adult cognitive function, muscle strength, and now pCMS in childhood.”

Thank you for the opportunity to improve this manuscript.

Sincerely,

Thomas V. Fernandez, M.D.

Associate Professor, Yale Child Study Center & Psychiatry

Vice Chair for Research, Yale Child Study Center

Co-Director, Tic Disorder/OCD/ADHD Clinic
